# Profiling of ginsenosides in the two medicinal *Panax* herbs based on ultra-performance liquid chromatography-electrospray ionization–mass spectrometry

**DOI:** 10.1186/s40064-016-3427-3

**Published:** 2016-10-12

**Authors:** Jia Liu, Yang Liu, Long Zhao, Zhong-Hua Zhang, Zhong-Hua Tang

**Affiliations:** The Key Laboratory of Plant Ecology, Northeast Forestry University, Harbin, 150040 China

**Keywords:** Ginsenosides, Part-specific, UPLC–MS, *P. ginseng*, *P. quinquefolius*

## Abstract

As the king of herb plants, ginseng has been used for nearly 5000 years in medicines in Asia and recently in the West. Ginsenosides, the main active constituents in *Panax* herbs, have prominent immunoregulatory effects. Although extensively studied in the roots, ginsenosides have not been studied with regard to their profiles and natural variations in the leaf, stem, petiole, lateral root, and main roots during development or among species. In this study, a sensitive ultra-performance liquid chromatography-electrospray ionization–mass spectrometry method with a shorter chromatographic running time was developed and validated for simultaneous quantification of ten ginsenosides. Comparing ginsenoside contents in various parts during different developmental stages revealed part-specific accumulation of most ginsenosides. Further investigation indicated that Rg3 accumulated at significantly higher levels in the petiole of *P. ginseng* than in that of *P. quinquefolius*. The relative ratio of ginsenoside Rb2 to Rb1 appears to be a candidate metabolic marker for identifying the ginseng cultivar within a diverse collection of ginseng accessions. In addition, the PCA showed that aboveground parts differed significantly between species and can be considered as species-specific markers rather than roots. This comprehensive survey, providing reliable, affordable and adequate scientific evidence, could be used to differentiate two species and discriminate ginseng cultivar ages.

**Electronic supplementary material:**

The online version of this article (doi:10.1186/s40064-016-3427-3) contains supplementary material, which is available to authorized users.

## Background

Ginseng (*Panax ginseng* C. A. Meyer), a perennial plant of the Araliaceae family, is a key component of traditional Chinese medicines (TCMs). It was discovered over 5000 years ago in China and today is still one of the most widely used botanicals in China, Korea and western countries (Wang et al. 1999). The genus *Panax* includes 3 species including *Panax ginseng* (Asian or Korean ginseng), *Panax notoginseng* (Chinese ginseng, also called Sanchi ginseng), and *Panax quinquefolius* (American ginseng) (Qi et al. 2011). In addition, there is considerable commercial interest in ginseng for food and health products. Recently, the root and rhizome of ginseng has been approved as a new resource food by the Ministry of Health of the People’s Republic of China. The roots of *P. ginseng* have diverse pharmacological effects, such as controlling blood pressure and stimulating the immune system, increasing learning and cognitive performance and antiaging, antioxidative, and anticancer activities (Christensen 2009; Keum et al. 2000). In northern China, the two major species are *Panax ginseng* and *Panax quinquefolius*, based on their morphological character and pharmacological function (Chen et al. 2008).

The primary biochemical and pharmacological activities of *Panax* plants have been attributed to ginsenosides (Leung and Wong 2010; Wang et al. 2010). Ginsenosides are triterpenoid saponins (or ginseng saponins) that are secondary metabolites almost exclusively produced in *Panax* species (Liang and Zhao 2008). Ginsenosides differ from each other in the number, linkage position and type of sugar moiety. To date, more than 150 naturally occurring ginsenosides have been identified from *Panax* plants (Zhao et al. 2015) and classified according to the aglycones ginsenoside skeleton into two major types: dammaranes and oleananes (Attele et al. 1999). The dammarane-type ginsenosides have three types of aglycone moieties: protopanaxadiol (PPD), protopanaxatriol (PPT), and ocotillol. The PPD-group saponins, such as ginsenosides Rb1, Rb2, Rc and Rd are glycosides, and each contains an aglycone with a dammarane skeletion, with sugar moieties attached to the β-OH at C-3 and/or C-20 (Table 1) (Yang et al. 2012). Ginsenosides Re, Rf, Rg1 and Rh1 belong to the PPT-type saponins, which consist of sugar moieties attached to the α-OH at C-6 and/or β-OH at C-20 (Fuzzati 2004). These ginsenosides commonly occur in *Panax* plants and are used in a wide range of pharmacological treatments for cancer, inflammation, allergy and hypertension etc., due to their have an immunomodulatory effect (Angelova et al. 2008; Song et al. 2012).

**Table 1:** Chemical structure of different types of ginsenosides and their classification based on the glycosides attached

Structure	Ginsenoside	R1	R2	R3	Formula
	Ginsenoside Rb1	–Glc^2_1^Glc		–Glc^6_1^Glc	C_54_H_92_O_23_
	Ginsenoside Rb2	–Glc^2_1^Glc		–Glc^6_1^Ara(p)	C_53_H_90_O_22_
	Ginsenoside Rc	–Glc^2_1^Glc		–Glc^6_1^Ara(f)	C_53_H_90_O_22_
	Ginsenoside Rd	–Glc^2_1^Glc		–Glc	C_48_H_82_O_18_
	Ginsenoside Rg3	–Glc^2_1^Glc		–H	C_42_H_72_O_13_
	Ginsenoside Rh2	–Glc		–H	C_36_H_62_O_8_
	Ginsenoside Rg1		–Glc^2_1^Rha	–Glc	C_42_H_72_O_14_
	Ginsenoside Re		–Glc^2_1^Glc	–Glc	C_48_H_82_O_18_
	Ginsenoside Rf		–Glc	–Glc	C_42_H_72_O_14_
	Ginsenoside Rh1		–Glc	–H	C_35_H_62_O_9_

*Glc* β-d-glucopyranosyl, *Ara(p)* α-l-glucopyranosyl, *Ara(f)* α-l-arabinofuranosyl, *Rha* α-l-rhamnopyranosyl

Structural characterization to identify ginsenosides in different *Panax* species has been highly developed using LC-MS/MS and nuclear magnetic resonance (NMR) (Li et al. 2000; Shergis et al. 2013; Xie et al. 2008; Yu et al. 2014). While NMR metabolite profiling provides a valuable metabolite signature of a complex sample, LC-MS has higher sensitivity and resolution, resolving individual chemical components into separate peaks (Nordstrom et al. 2006). Among the various LC platforms, the soaring development of column particle materials has highlighted a new addition to chromatographic separation technology, ultra-performance liquid chromatographic (UPLC) separation technology, which allows satisfactory separation, good resolution and sensitivity, and high-speed detection for complex biological samples such as herbal medicines (Xie et al. 2008). An HPLC-based metabolic profiling and quantitative method has been developed for quality control of leaves of different *Panax* plants (Yang et al. 2013). Furthermore, UPLC analysis showed that the main roots of *Panax ginseng* and *P. quinquefolius* differ significantly in their levels of six ginsenosides (Xie et al. 2008). Nevertheless, comprehensive profiling of part and developmentally specific accumulation of ginsenosides in ginseng has received little attention. In addition, because the length of the detection time needed for these methods interferes with the active components of the sample, a method with a shorter detection time needs to be developed.

In the present study, an integrated approach combining a UPLC assay with electrospray ionization–mass spectrometry was developed to identify part-specific, developmentally controlled accumulation of ginsenosides and determine whether the two main species of ginseng in northern China differentially accumulate ginsenosides. Our data showed that the newly established method is accurate, rapid and discriminatory for identifying the different species and predicting the ages of the ginseng samples.

## Methods

### Materials and reagents

All chemicals were of analytical grade. Acetonitrile and methanol (J & K Scientific, Beijing, China) were of UPLC-grade. Deionized water was purified with a Milli-Q Academic ultra-pure water system (Millipore, Milford, MA, USA). Ginsenoside Rg1, Re, Rb1, Rb2, Rc, Rd, Rf, Rh1, Rh2 and Rg3 were purchased from Beijing Science and Technology (Beijing, China). The purities of these standards were higher than 98 % according to HPLC analysis. Standard stock solutions were prepared using methanol as a solvent and stored at −20 °C. Standard solutions of ginsenosides were prepared just before use by mixing individual stock solutions and diluting these mixtures with methanol.

### Plant materials

**Fig. 1:**
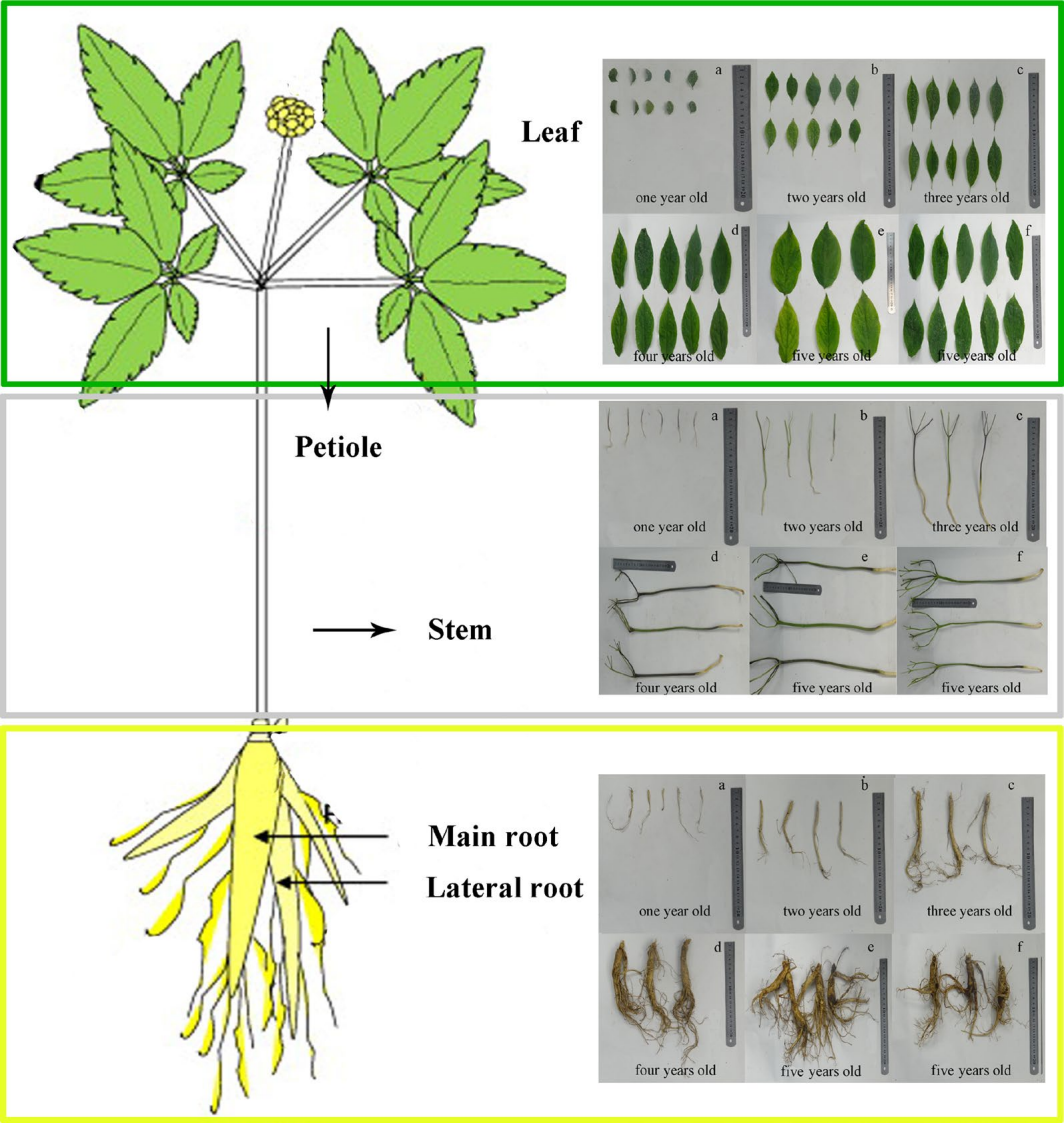
Schematic illustrations of collected parts of *Panax ginseng* and *P. quinquefolius*. **a**–**e**. *P. ginseng*: **a** 1, **b** 2, **c** 3, **d**, 4, **e** 5 years old. **f** *P. quinquefolius*: 5 years old

The ginseng core species used in this study were from the Changbai Mountain field base located in northeastern China (42°01′–43°24′ N, 127°48′–129°11′ E). Roots, lateral roots, petioles, stems, and leaves at different developmental stages were collected from 1-, 2-, 3-, 4-, 5-year *Panax ginseng* and 5-year *Panax quinquefolius*, resulting in 90 individual samples (Fig. 1). For each part, three samples were obtained for analysis by mixing six individual samples together. All the samples were harvested at 9:00 and 11:00 on 15 August 2015. Each group of plant part was carefully washed, cut into small pieces, and stored at −70 °C until vacuum freeze-drying. Samples were taken from three plants per line and pooled for each biological replicate.

### Sample preparation and extraction

Analysis of ginsenosides compounds in *Panax ginseng* and *Panax quinquefolius* were performed combined with previous methods (Liu et al. 2016; Yu et al. 2014). In order to improve the extraction efficiency for ginsenoside, this extraction method was optimized. 1.0 g of each part was weighed, extracted overnight in a Soxhlet extractor and at 4 °C with 60 mL precision water-saturated *n*-butanol. The next day, the extract solution was filtered with a 0.2 μm syringe filter (Millipore Co., Bedford, MA), the filtrate was separated with a funnel and washed with 20 mL 2 % w/v NaOH solution. The upper solutions were combined and evaporated to dryness, then diluted to the desired volume with methanol. Following centrifugation at 10,000×*g* for 15 min, the extracts were absorbed and filtered through a 0.45 μm syringe filter before UPLC analysis.

### Chromatographic conditions

Samples were separated at 25 °C using an Ultra-Performance LC (UPLC) system (Waters, Japan) with a LC-20AD pump, a temperature controller, column oven, SIL-20A autosampler (Waters, Japan), and ACQUITY UPLC BEH C_18_ Column (1.7 µm, 2.1 mm × 50 mm) with an in-line filter. The mobile phase consisted of (A) water and (B) acetonitrile in the elution program optimized as follows: 30 % B (0–4.5 min), 30–60 % B (4.5–6 min), 60–90 % B (6–6.5 min), 90 % B (6.5–7.5 min), 90–30 % B (7.5–8 min), 30 % B (8–10 min). The flow rate was 0.25 mL/min, and the injection volume was 5 μL.

### Mass spectrometry conditions

Tandem mass spectrometry was performed using an QTRAP 5500 Ion Trap Mass Spectrometer (AB SCIEX, USA) equipped with an electrospray ionization (ESI) source in the positive ion detection mode. MS source conditions were set as follows: ion spray voltage, 5500 V; turbo spray temperature, 500 °C; high purity nitrogen was used in all units; nebulizer gas, 25 psi; curtain gas, 20 psi. In this study, the level of ginsenosides observed in positive ion mode was higher than that in negative ion mode, so the positive ion mode was selected. Samples were quantified from positive multiple reactions, monitoring the *m/z* for Rg1, Re, Rd, Rb1, Rb2, Rf, Rh1, Rh2, Rg3, and Rc that gave 824.0, 970.0, 964.1, 1131.1, 1101.8, 801.7, 639.2, 623.2, 786.1, and 1101.2, respectively. During infusion of each of the standards, the collision energy was varied from 12 to 83 V to find the optimal collision energy. The most abundant fragment ions for each ginsenoside were chosen for the multiple reaction monitoring. The optimal conditions for the mass spectrometry, for fragment ions and the main fragment ions of each ginsenoside gave 824.0 → 643.9 for Rg1, 970.0 → 789.9 for Re, 964.1 → 767.2 for Rd, 1131.1 → 789.2 for Rb1, 1101.8 → 335.0 for Rb2, 801.7 → 424.2 for Rf, 639.2 → 621.1 for Rh1, 623.2 → 605.2 for Rh2, 786.1 → 325.0 for Rg3, and 1101.2 → 334.4 for Rc. The declustering potential and collision energy were 70/61 for Rg1, 125/67 for Re, 111/17 for Rd, 120/83 for Rb1, 90/81 for Rb2, 40/26 for Rf, 50/12 for Rh1, 160/13 for Rh2, 60/15 for Rg3 and 149/76 for Rc. Data were collected using Analyst 1.4.2 software (AB SCIEX, Canada).

### Statistical analyses

Ten ginsenosides were used in a hierarchical clustering analysis in the program R (www.r-project.org/) to study ginsenoside part-specific accumulation. Results were subjected to an analysis of variance (ANOVA) to determine any significant differences between differential species. If the ANOVA showed any significant differences, then Duncan’s honestly significant difference (HSD) post hoc test was used to determine any differences between individual treatments (SPSS 17.0, SPSS, USA). Principal component analysis was used to compare the development stage and different species according to the ginsenoside profiles of different parts.

## Results

### Establishment of UPLC/MS method for ginsenoside analysis

Owing to the complexity of metabolites, many components may be coeluted during analyses. To develop a sensitive and accurate UPLC/MS method for determining the active compounds in ginseng, we used a triple quadruple mass spectrometer equipped with ESI, one of the most useful tools available for simultaneous quantification of ginsenosides, to analyze the compounds in ginseng. To quantify the analytes using the MRM mode, we evaluated full scan and product ion spectra of the analytes. First, in tests of positive and negative ion detection modes, the positive ESI achieved higher sensitivities than the negative. With positive ESI, Rd, Rf, Rh1, Rh2, and Rg3 formed predominately protonated molecules, [M + H]^+^, at *m/z* 964.1, 801.7, 639.2, 623.2, and 786.1, respectively, in the full-scan spectra. In addition, Rg1, Re, Rb1, Rb2, and Rc predominantly formed sodium adduct ions, [M + Na]^+^, at *m/z* of 824.0, 970.0, 1131.1, 1101.8, and 1101.2 with the response approximately 5 times higher than with [M + H]^+^. The [M + H]^+^ and [M + Na]^+^ ions were therefore chosen as the precursor ions to obtain their major fragment ions for MRM analysis. The fragment ions at *m/z* values of 643.9, 789.9, 767.2, 334.4, 621.1, 605.2, 789.2, 335.0, 325.0, and 424.2, were present in the highest abundance for Rg1, Re, Rd, Rc, Rh1, Rh2, Rb1, Rb2, Rg3, Rf, respectively (Additional file 1: Figure S1). The instrument was tuned to yield the maximum product ion for each compound.

In optimizing the UPLC system to detect the 10 ginsenosides, chromatographic separation was tested on several C_18_ columns to achieve the best efficiency and peak shape. The ACQUITY UPLC BEH C_18_ column (1.7 μm, 2.1 mm × 50 mm) gave good peak shape when ultra-pure was used as the mobile phase. Different mobile phases were evaluated to improve LC separation and enhance MS sensitivity. When methanol and acetonitrile were tested as the organic modifier, acetonitrile was superior for ionizing most ginsenosides. In tests on the isocratic and gradient systems, the gradient system achieved better peak shape than the isocratic system; thus, a gradient elution with a mobile phase consisting of acetonitrile and no acid in water gave optimal peak shape and mass spectral response for the analytes.

**Fig. 2:**
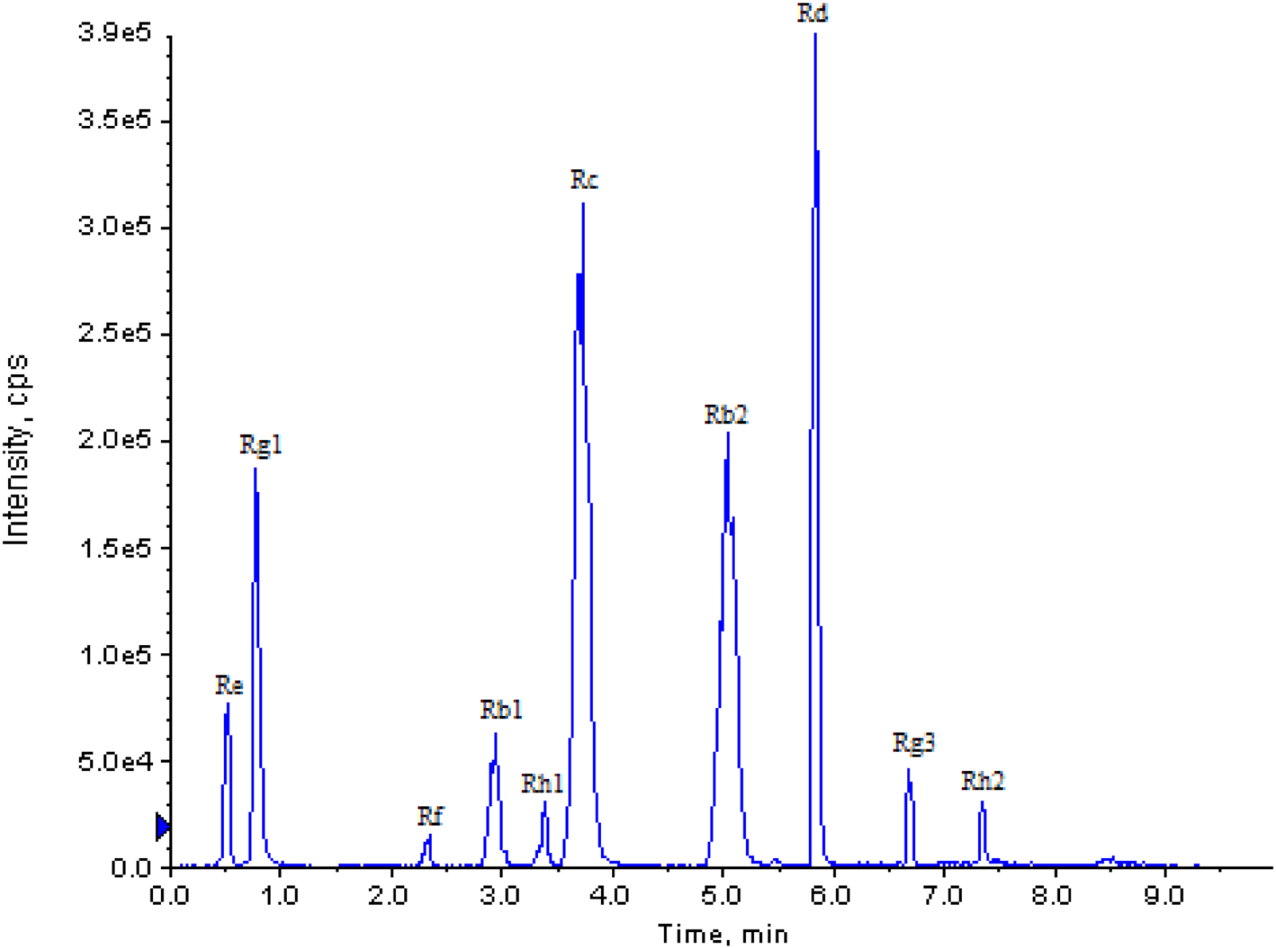
UPLC chromatographic fingerprints of 10 standard solutions at 1 min Rg1 (10 min separation on a 1.7 µm, 2.1 mm × 50 mm ACQUITY UPLC BEH C_18_ column)

The representative total ion current (TIC) spectra in 10 min was obtained from the analysis (Fig. 2). The retention time of Re, Rg1, Rf, Rb1, Rh1, Rc, Rb2, Rd, Rg3, and Rh2 was 0.76, 0.78, 2.32, 2.97, 3.48, 3.69, 4.94, 5.83, 6.69, and 7.34 min, respectively. No endogenous interference was detected at these retention times for the 10 analytes; therefore, high, very acceptable selectivity was achieved by this method. Regression equations and a linear range for calibration curves were also obtained. To avoid bias to the low concentrations of the standard curve caused by the high concentrations, the calibration curves were separated for different ranges. Within the linear range, the calibration curve had good linearity (*r* ^2^ > 0.999) for each analyte (Additional file 2: Table S1). In addition, the proposed method delivers reliable accuracy and good reproducibility for the simultaneous separation and detection of the metabolites using UPLC/MS (Additional file 2: Table S2, 3). The optimized method was employed for the determination of the ginsenosides by comparing the area calculated for each peak to the standard curves obtained from the authentic ginsenoside standards (Additional file 2: Table S1). Calibration curves were plotted using five concentrations of each ginsenoside standard according to the peak area.

### Specific accumulation of ginsenosides in various parts of *P. ginseng*

**Fig. 3:**
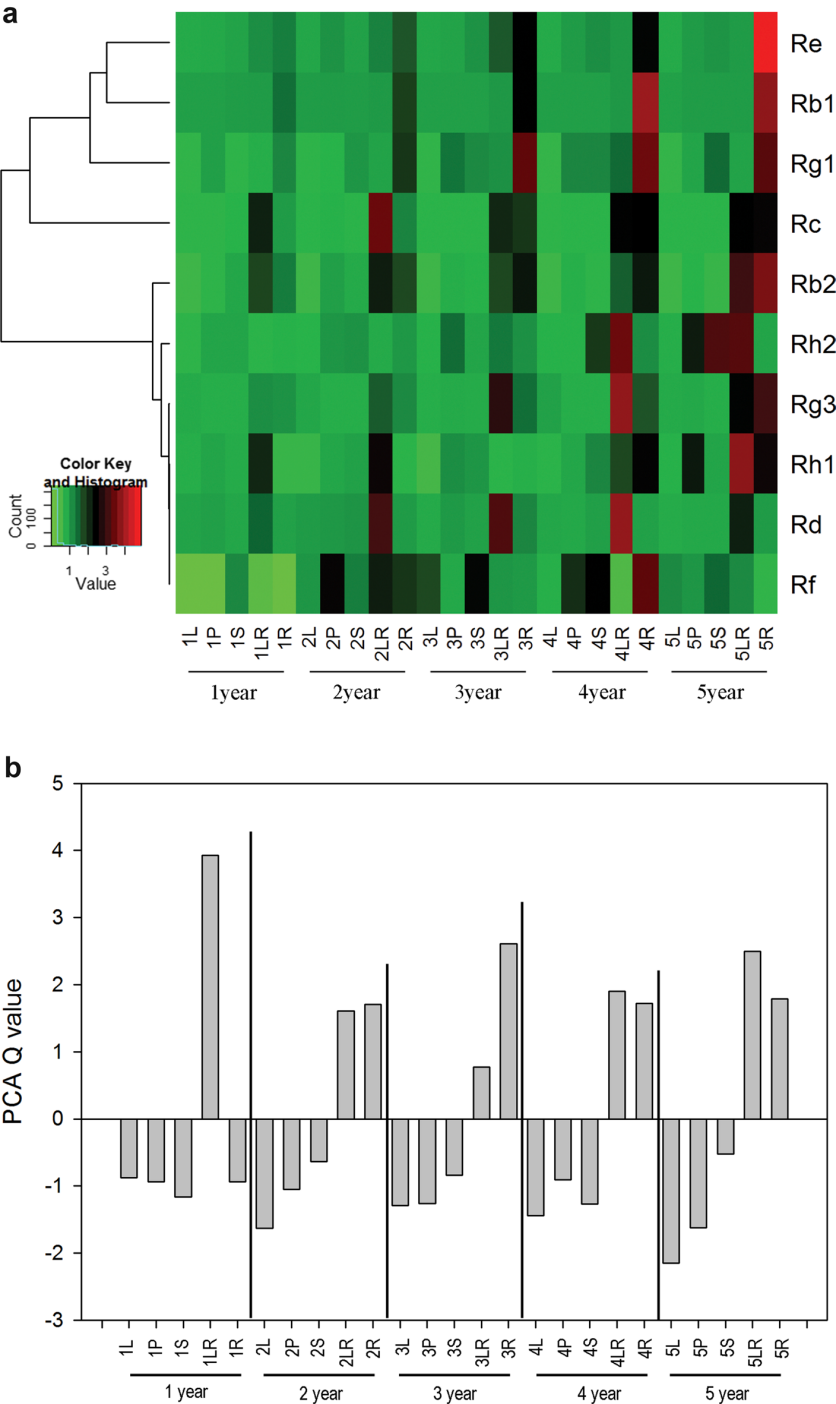
Distribution of ginsenosides in leaf, stem, petiole, lateral root, and main root samples of *P. ginseng* at different growth stages. Heat map visualization of relative differences of ginsenosides in different growth stages of five parts. **a** Content of each ginsenoside, normalized for linkage hierarchical clustering. Each part type is visualized in a *single column*, and each ginsenoside is represented by a *single row*. *Red* indicates high relative abundance; *green* is low relative abundance (see *color key* above the heat map). **b** Score plot for principal component analysis. Negative values are below the average

To investigate the accumulation of ginsenosides in different part of *P. ginseng*, samples from five different parts were collected, including leaf, petiole, stem, lateral root, and main root. For each part, samples from different growth stage were obtained. The profile for the main ginsenosides was visualized using a hierarchical cluster analysis (HCA; Fig. 3). Accumulation of the ginsenosides displayed a clear phenotypic variation in terms of their abundance in different parts of the same age. Roots contained the highest levels of most ginsenosides, followed by lateral roots, stems, and petioles; leaves had the lowest. In addition to the greatest accumulation of ginsenosides, roots also had the most complex profile for ginsenoside composition in the different ages of the parts.

Based on their part- and growth stage-specific accumulation patterns, ginsenosides could be clearly grouped into five stages (Fig. 3a). Ginsenosides in the first year had higher levels in lateral roots than in other parts and were mainly represented by PPD-type ginsenosides, including some of the major ginsenosides such as Rc, Rb1, and Rd. In the second year, the PPD-type such as Rc, Rb2, Rg3, and Rd predominated, again with higher levels in lateral roots. Moreover, in the same stage, other ginsenosides had their highest levels in the main roots and were mainly represented by PPT-type, Re, Rg1, and Rf. Major PPD- (Rc, Rb2, Rd, and Rg3) and PPT-types (Re and Rg1) were tightly grouped in lateral roots and main roots, respectively, during the third year. Three PPD-types, including Rc, Rh2 and Rg3, in the fourth year were significantly higher in lateral roots than in leaves, petioles, stems and main roots. Furthermore, one PPD-(Rb1) and two PPT-types (Rg1 and Rf) significantly accumulated in the main roots. Re and Rg1 during the fifth year had highest levels in the main roots and were mainly represented by PPT, which have been characterized for their medicinal value. However, the PPD-type Rh2 had higher levels in lateral roots, followed by stems, petioles, main roots and leaves. In addition, PPD-types Rb1, Rb2 and Rg3 showed the same accumulation pattern as the PPT-types Re and Rg1, suggesting highest accumulation in the main roots than in other parts during the fifth year.

In addition, the “Q” score of the principal component is an indicator of a comprehensive, scientific evaluation of objective phenomenon, which has no practical significance. In the Fig. 3b, the comprehensive PCA Q value result revealed that the overall accumulation of ginsenosides in different parts and stages followed a relatively stable distribution. The above- and belowground parts could be clearly differentiated, except for the roots of 1-year olds. Leaves contained the lowest levels of ginsenosides, followed by petioles and stems, and second year and fifth year parts shared this uniform tendency, which these ginsenosides in leaves had lower levels than in the petioles and stems. Contrasting the aboveground parts ginsenosides changing, we observed that the belowground parts (lateral roots and main roots) increasing their concentration in main roots is higher than those increasing their concentration in lateral roots of 2- and 3-year-old plants. In addition, an increase of the number of Q value which accumulate higher in lateral roots than main roots during 4 to 5-year-old samples.

### Ginsenoside accumulation patterns during development

**Fig. 4:**
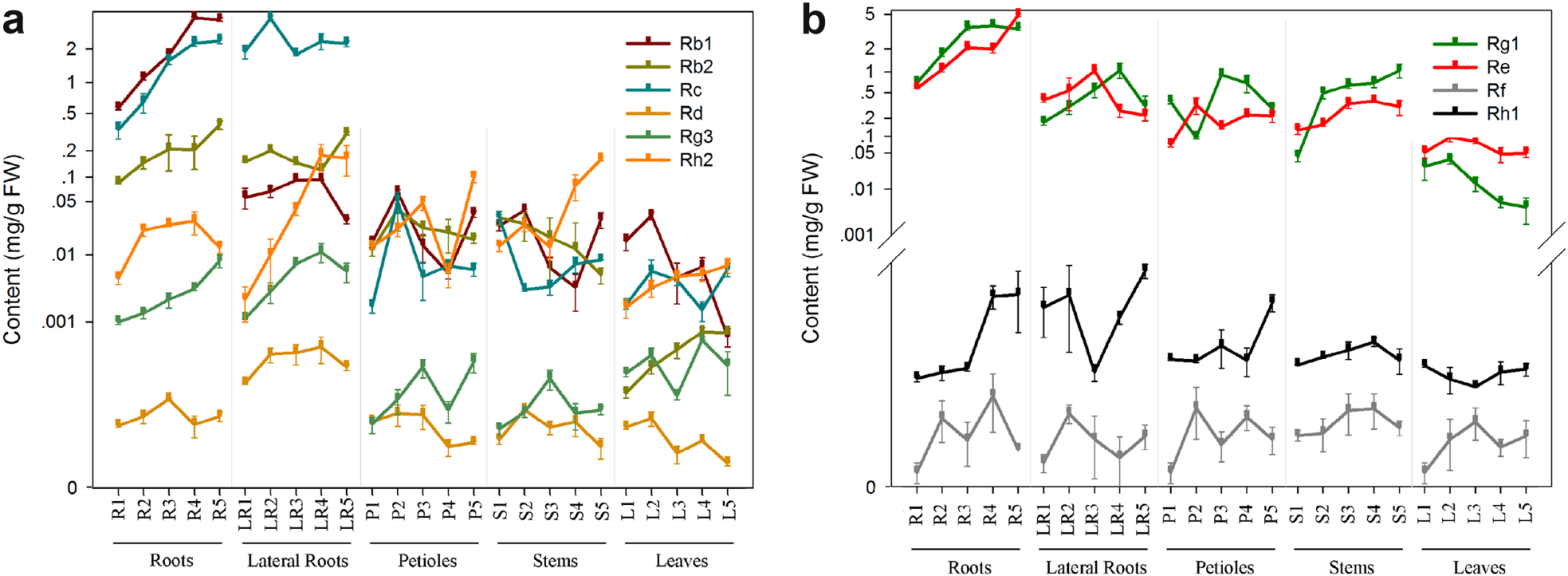
Accumulation patterns of different ginsenosides in main roots, lateral roots, petioles, stems, and leaves at various developmental stages of *Panax ginseng*. **a** Protopanaxadiol-type ginsenosides. **b** Protopanaxatriol-type ginsenosides. *FW* fresh mass. The full name of parts abbreviations R1, 2, 3, 4, 5 are main roots 1 year old, 2 years old, 3 years old, 4 years old, 5 years old; LR1, 2, 3, 4, 5: lateral roots 1, 2, 3, 4, 5 years old; P1, 2, 3, 4, 5: petioles 1, 2, 3, 4, 5 years old; S1, 2, 3, 4, 5: stems 1, 2, 3, 4, 5 years old; L1, 2, 3, 4, 5: leaves 1, 2, 3, 4, 5 years old. *Each bar* represents the mean ± SEM, *n* = 3

To further clarify ginsenoside accumulation patterns during different developmental stages, five parts were sampled at five stages, and the 10 major ginsenosides in each part were quantified. Ginsenosides with different modifications accumulated differently at different developmental stages. The various forms of ginsenosides were subsequently quantified, and the major ginsenosides were selected to determine the range of variations observed in leaves, petioles, stems, lateral root and main roots during each stage (Fig. 4).

Most PPD-types such Rb1, Rb2, Rc and Rg3 significantly increased in the roots during years 1–5 (R1–R5) (Fig. 4a). The levels of Rb1 and Rb2, showed marked cross-change during the lateral root stage (LR2–LR5), sharply increasing at early stages (P1–P2). Levels of Rb2 decreased over time in stems (S1–S5). Rb1 and Rd sharply decreased and Rb2 and Rh2 levels increased in leaves during later stages (L2–L5). For most of the PPT-types such as Rg1, Re and Rh, accumulation in roots significantly increased during R1 to R5 (Fig. 4b). In lateral roots, Rg1 and Re sharply increased during LR1 to LR3. However, Rg1 sharply decreased during LR4 to LR5 and for Re during LR3–LR5. In addition, the level of Rg1 and Re had an inverse relationship during all petiole stages (P1–P5). Re and Rf increased in a similar way in stems (S1–S5). In contrast, a discrepant synthesis of Re and Rh1 during leaf development (L1–L5) suggested that their accumulation was developmentally controlled.

### Differential accumulation of ginsenosides between *P. ginseng* and *P. quinquefolius*

**Fig. 5:**
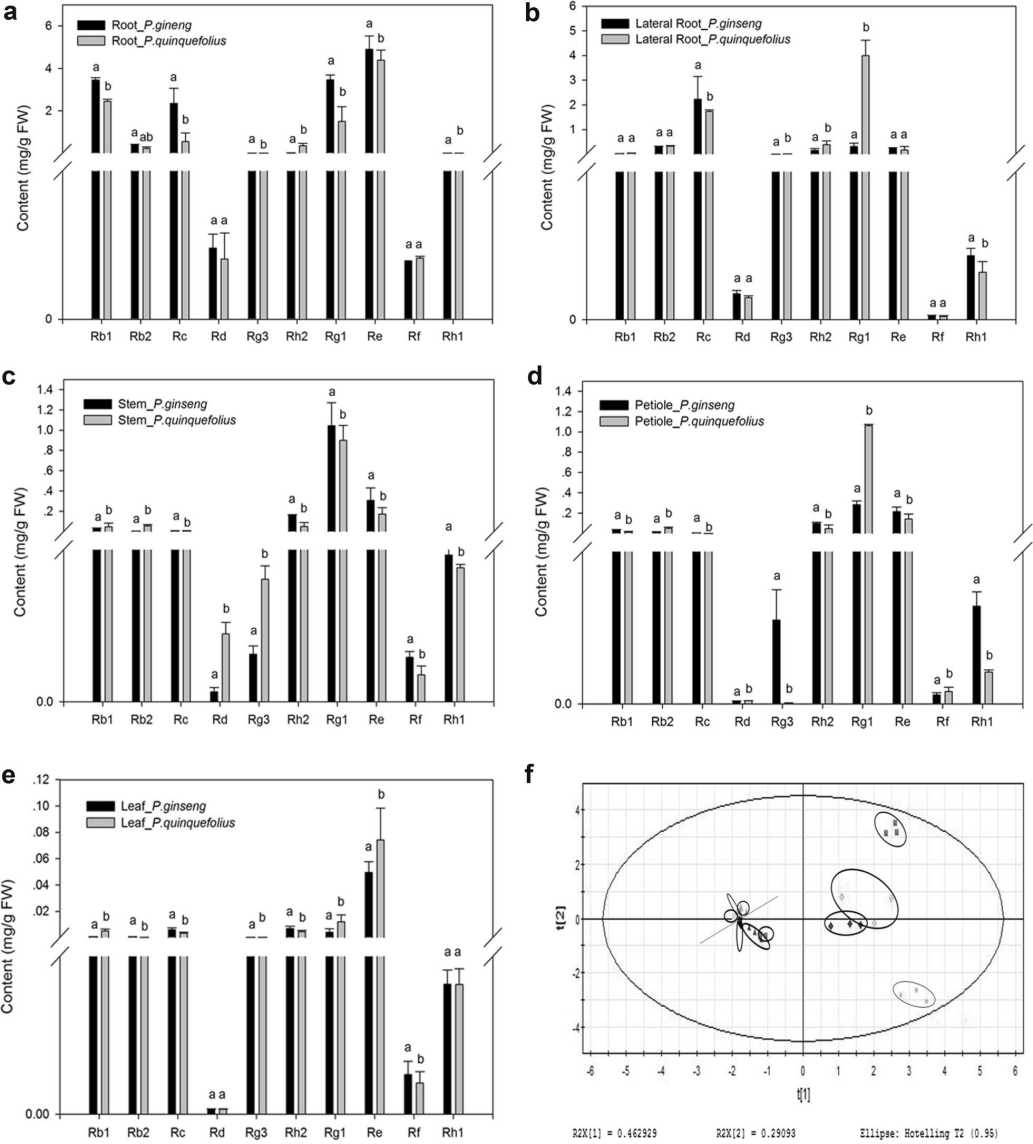
Differential accumulation of ginsenosides in main roots, lateral roots, petioles, stems, and leaves between *Panax ginseng* and *P. quinquefolius*. **a** Main roots. **b** Lateral roots. **c** Stems. **d** Petioles. **e** Leaf. *FW*, fresh mass. **f** Two-dimensional diagrams of PCA1 and PCA2. For *Panax ginseng*: *red* main root, *blue* lateral root, *purple* stem, *light blue* petiole, *yellow* leaf. For *P. quinquefolius*: *green* main root, *dark yellow* lateral root, *dark green* stem, *dark red* petiole, *black* leaf. Mean values with the *same letter* within a *column* did not differ significantly according to Duncan’s multiple comparison test with a family error rate of 0.05. Each *bar* represents the mean ± SEM, *n* = 3

Differences in genetic background and geographical distribution between *P. ginseng* and *P. quinquefolius* may lead to differing accumulation and composition of ginsenosides in these two species. For each part sample (leaf, petiole, stem, lateral root, main root), the relative concentration of each ginsenoside of interest was determined for *P. ginseng* and *P. quinquefolius* (Fig. 5).

In the main roots, the levels of seven ginsenosides differed significantly *between the two species.* Among them, four PPD-types (Rb1, Rb2, Rc, Rg3) were higher in *P. ginseng* than in *P. quinquefolius*. Three major PPT-types (Rg1, Re, Rh1) were higher in *P. ginseng* than in *P. quinquefolius* (Fig. 5a), but Rg3, Rg1, and Rh2 in lateral roots were lower in *P. ginseng* than in *P. quinquefolius* (Fig. 5b). In stems, major PPD-types Rb1, Rb2, Rd, and Rg3 were significantly elevated levels in *P. quinquefolius* compared with *P. ginseng* (Fig. 5c). In petioles, Rg3 was 80 times higher and Rh1 was 3 times higher in *P. ginseng* than in *P. quinquefolius* (Fig. 5d). Similarly, Rb2, Rc, Rf, and Rh2 levels in leaves were higher in *P. ginseng* than in *P. quinquefolius*, whereas Rb1, Rg3, Rg1 and Re were significantly higher in *P. quinquefolius* than in *P. ginseng* (Fig. 5e). No significant difference was found between the two species for Rd and Rh1. Furthermore, a two-dimensional PCA score plot (Fig. 5f) was able to discriminate the differential levels in parts between *P. ginseng* and *P. quinquefolius*, thus simplifying data management. The above results suggested that the levels of ginsenosides in the two *Panax* species could be determined through UPLC coupled with PCA and the values of ginsenosides.

## Discussion

As compared with simultaneous determination of several ginsenosides in *P. ginseng*, a metabolite profiling study requires higher resolution and sensitivity. The UPLC-ESI–MS system used here provides a rapid, effective, and convenient profiling analytical method for a wide range of ginsenosides present in *P. ginseng*. Compared with previous HPLC and UPLC research hampered by run times as long as 70 and 21 min (Li et al. 2005; Wu et al. 2015; Yang et al. 2013), the major ginsenosides were excellently separated with superior peak shapes, especially the 10 saponins quantified and qualitated during a 10-min run. In this method, ginsenosides with various modifications were in this study. Coinciding with previous reports on roots of *P. ginseng* and *P. quinquefolius*, PPD and PPT-type ginsenosides were found. Besides, the method can be used to differentiate *P. ginseng* and *P. quinquefolius*, especially to distinguish their cultivation stage and species. In addition, ginsenosides are used in biological/pharmacological experiments, thus the understanding of the levels in different parts and different *genus* is of largely importance. Often there is inconsistency in biological activities of ginsenosides, such as anti-cancer (Rg1, Rg3 and Rb1), mmunomodulatory (Re), neuroprotective (Rf), radioprotective (Rh1), hypoglycemic activities (Rb2) and anti-stress properties (Rc and Rd) (Cho 2012; Joo et al. 2010; Shibata 2001; Zhao et al. 2011). Therefore, a comprehensive analysis of specific parts and developmental stages and to identify the species and age are essential for ginseng.

The levels of various ginsenosides in the roots of the *Panax* species could be used to identify the stage of development (Dan et al. 2009; Mao et al. 2014). A rapid examination of the qualitative and quantitative differences in the ginsenosides present in various parts of ginseng at different developmental stages allowed a comprehensive profiling and a better understanding of the distribution of the active ingredients within the plants. In addition to having the highest amount of ginsenosides, the roots also yielded the most complex profile of ginsenosides in the different parts, suggesting potential diversity in the pharmacological components in other parts of the plants, such as saponins in the leaves. Ginsenosides not only increase immune system, adjust the body’s function, and promote metabolism, and affects a wide range of biological activities in the nervous system, cardiovascular system, and immune system, but also have been widely used in clinical practice (Shan et al. 2014). Previous investigations on leaves of *P. ginseng* identified 37 compounds using UHPLC-QTOF-MS/MS (Mao et al. 2014) and suggested that ginsenosides provide the main medical activity in ginseng leaves. While quantitatively analyzing ginsenosides in leaves, we found that Rb2 and Rh2 accumulate during leaf growth, suggesting possible roles of Rb2 and Rh2 in ginseng leaf growth and medical efficacy, since Rh2 is a key component in the inhibition of cancerous cells (Liu et al. 2015b). In developing ginseng roots, some ginsenosides steadily accumulate peaking in older roots (Liu et al. 2015a). In addition, our study showed that all types of ginsenoside were higher in the roots than in other parts, thus explaining why the roots are the major source of drugs. Thus, based on our evaluation of the profiles of different ginsenosides, the accumulation of ginsenosides in ginseng is part-specific and developmentally dependent, further suggesting their widespread spatial distribution in the plants and a special use for determining the age of the parts.

In addition, some ginsenosides differentially accumulated between *P. ginseng* and *P. quinquefolius*, suggesting that the ratio of Rb2 to Rb1 could be used as a biomarker to discriminate *P. ginseng* from *P. quinquefolius*. A ratio less than 0.4 for Rb2/Rb1 in roots is indicative of *P. quinquefolius*; a significantly higher value indicates *P. ginseng* (Yuan et al. 2010). In the present study, the Rb2/Rb1 ratio in *P. quinquefolius* was 0.05 and 1.1 in *P. ginseng* leaves. However, the ratio of Rb2/Rb1 followed an opposite trend in stems with a ratio of 1.2 in *P. ginseng* and 0.1 in *P. quinquefolius*. Another significant factor was the level of the ginsenoside Rg3 in *P. ginseng*, which was nearly 80 times higher than in the petiole of *P. quinquefolius* (Fig. 5d). These results suggested that the Rb2/Rb1 ratio in roots, leaves, and stems and the Rg3 content are good candidate metabolic markers for identifying the ginseng species within a diverse collection of ginseng accessions. In addition, the stems, leaves, and petioles of plants can be sampled to make a cleared significant distinction (Fig. 5f), so the valuable roots do not need to be sacrificed. The differential accumulation of ginsenosides between *P. ginseng* and *P. quinquefolius* further establish the basis for the differences in the medical efficacy of the two species.

## Conclusions

The highly sensitive UPLC–MS method was developed in the present study to simultaneously determine the 10 ginsenosides (Rg1, Re, Rb1, Rb2, Rc, Rd, Rf, Rh1, Rh2 and Rg3) in two ginseng species compromising the merits of selectivity, detection speed, precision, accuracy, and extraction recovery. The analysis of different parts at different developmental stages is thus fast and easy due to the relatively short chromatographic running time. This study is the first comprehensive analysis of ginsenosides with main concern on part type and developmental stage in the two major species of *Panax*, *P. ginseng* and *P. quinquefolius*. Rg3 accumulated at significantly higher levels in the petiole of *P. ginseng* than in that of *P. quinquefolius*. The relative ratio of ginsenoside Rb2 to Rb1 appears to be a candidate metabolic marker for identifying the ginseng cultivar within a diverse collection of ginseng accessions. On the other hand, through aboveground parts to discriminate age and species, we can preserve the great medicinal value roots. Hence, this work is of great importance for authenticate commercial ginseng samples regardless of the species, plant parts and ages, ultimately this strategy is promising for revealing and elucidating the metabolic outcomes as a result of plant origin, parts, growth stage and different cultivation methods.

## Additional files

10.1186/s40064-016-3427-3 The product ion scan spectra of the ginsenosides: (a) Rg1; (b) Re; (c) Rd; (d) Rc; (e) Rb1; (f) Rb2; (g) Rf; (h) Rg3; (i) Rh1; (j) Rh2.

10.1186/s40064-016-3427-3 The regression equations, linear range, LODs and LLOQs the tested compounds. **Table S2**. The intra-day and inter-day precision, accuracy and repeatability analysis of Ginsenoside Rg1, Re, Rb1, Rb2, Rc, Rd, Rf, Rh1, Rh2 and Rg3 at low, medium, and high concentration levels (n=6). **Table S3**. Recovery and matrix effect of bioactive ginsenosides in plants (n = 6).
