# Supplementary figures and images for: Profiling of ginsenosides in the two medicinal Panax herbs based on ultra-performance liquid chromatography-electrospray ionization–mass spectrometry
Source: Springerplus. 2016 Oct 12;5(1):1770. doi: 10.1186/s40064-016-3427-3 (PMC5059545; doi:10.1186/s40064-016-3427-3)

Figure S1. The product ion scan spectra of the ginsenosides: (a) Rg1; (b) Re; (c) Rd; (d) Rc; (e) Rb1; (f) Rb2; (g) Rf; (h) Rg3; (i) Rh1; (j) Rh2.


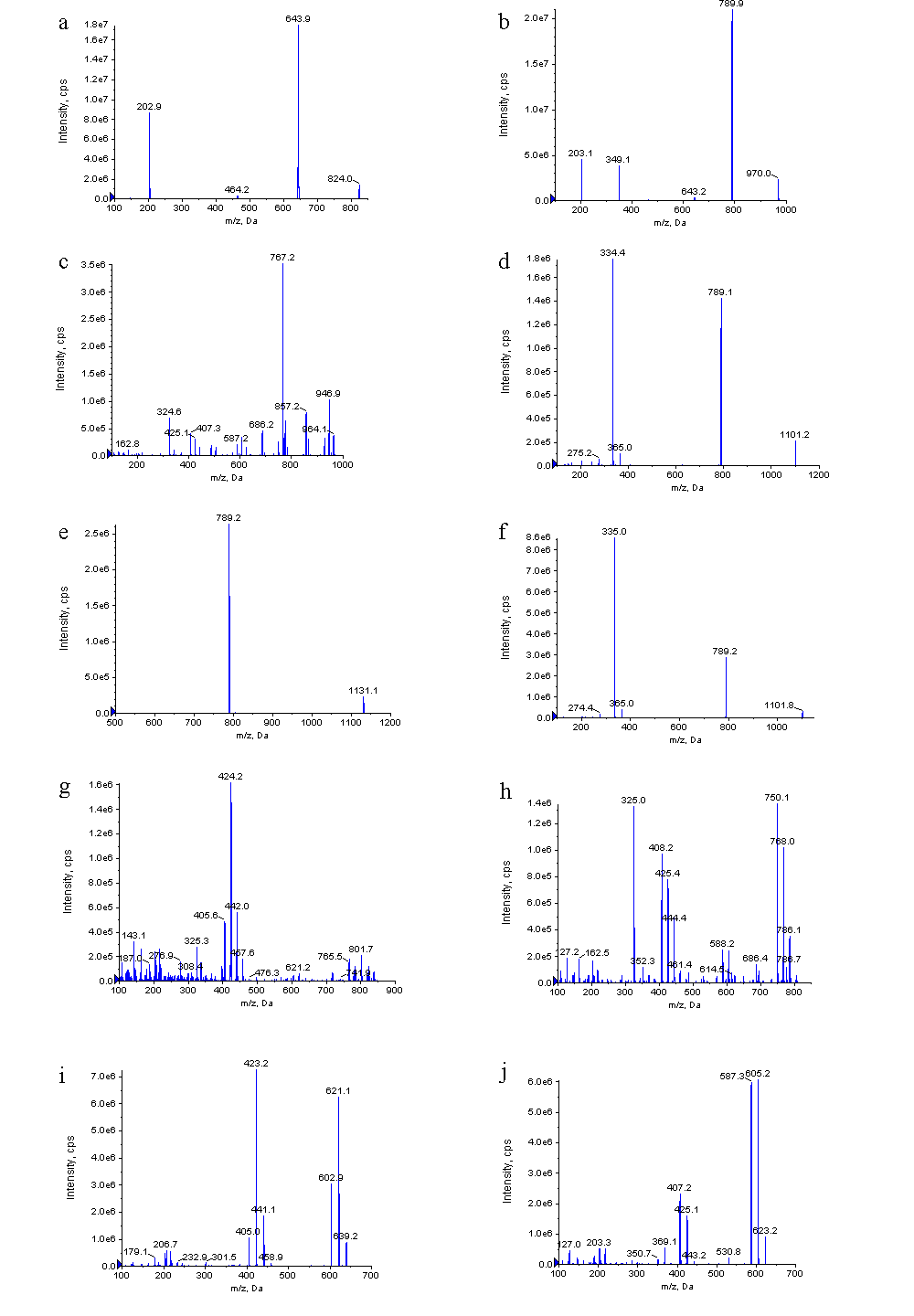

Supplement: Supplementary file 1 — 10.1186/s40064-016-3427-3 The product ion scan spectra of the ginsenosides: (a) Rg1; (b) Re; (c) Rd; (d) Rc; (e) Rb1; (f) Rb2; (g) Rf; (h) Rg3; (i) Rh1; (j) Rh2. [file 40064_2016_3427_MOESM1_ESM.doc]
