# Supplementary material for: Profiling of ginsenosides in the two medicinal Panax herbs based on ultra-performance liquid chromatography-electrospray ionization–mass spectrometry
Source: Springerplus. 2016 Oct 12;5(1):1770. doi: 10.1186/s40064-016-3427-3 (PMC5059545; doi:10.1186/s40064-016-3427-3)
Supplement: Supplementary file 2 — 10.1186/s40064-016-3427-3 The regression equations, linear range, LODs and LLOQs the tested compounds. Table S2. The intra-day and inter-day precision, accuracy and repeatability analysis of Ginsenoside Rg1, Re, Rb1, Rb2, Rc, Rd, Rf, Rh1, Rh2 and Rg3 at low, medium, and high concentration levels (n=6). Table S3. Recovery and matrix effect of bioactive ginsenosides in plants (n = 6). [file 40064_2016_3427_MOESM2_ESM.doc]

Table S1. The regression equations, linear range, LODs and LLOQs the tested compounds

| Ginsenosides | Range (ng/mL) | Calibration curves | Correlation coefficient (*r*) | Limit of detection (ng/mL) | Limit of quantitation (ng/mL) |
| --- | --- | --- | --- | --- | --- |
| Rg1 | 1-10000 | Y=14044x+597153 | 0.9999 | 0.26 | 1 |
| Re | 1-10000 | Y=10290x+46314 | 0.9997 | 0.26 | 1 |
| Rf | 0.1-1000 | Y=3821.7x+2931.5 | 0.9991 | 0.026 | 0.1 |
| Rh1 | 0.1-1000 | Y=31047x+15917 | 0.9995 | 0.026 | 0.1 |
| Rc | 1-10000 | Y=65238x+309468 | 0.9998 | 0.26 | 1 |
| Rd | 0.1-1000 | Y=907112x+4074.8 | 0.9997 | 0.026 | 0.1 |
| Rb1 | 1-10000 | Y=40057x+112176 | 0.9996 | 0.26 | 1 |
| Rb2 | 1-10000 | Y=155693x+239194 | 0.9995 | 0.26 | 1 |
| Rh2 | 0.1-1000 | Y=1189.3x+42122 | 0.9997 | 0.026 | 0.1 |
| Rg3 | 0.1-1000 | Y=6531.4x+20272 | 0.9998 | 0.026 | 0.1 |

Table S2. The intra-day and inter-day precision, accuracy and repeatability analysis of Ginsenoside Rg1, Re, Rb1, Rb2, Rc, Rd, Rf, Rh1, Rh2 and Rg3 at low, medium, and high concentration levels (n=6).

| Ginsenosides | Nominal concentrations (ng/mL) | Intra-day | | Inter-day | | Repeatability (relative standard deviation %) |
| --- | --- | --- | --- | --- | --- | --- |
|  |  | Precision (relative standard deviation %) | Accuracy (relative error %) | Precision (relative standard deviation %) | Accuracy (relative error %) |  |
| Rg1 | 50 | 2.08 | -0.67 | 1.69 | -1.00 | 1.79 |
|  | 500 | 0.27 | 0.03 | 0.47 | -0.07 | 0.30 |
|  | 5000 | 0.78 | 0.17 | 0.91 | -0.22 | 0.80 |
| Re | 50 | 1.03 | 0.67 | 1.69 | -1.00 | 1.66 |
|  | 500 | 0.87 | -0.27 | 1.28 | 0.13 | 0.91 |
|  | 5000 | 0.52 | -0.12 | 0.67 | -0.36 | 0.37 |
| Rf | 5 | 1.66 | 1.00 | 3.29 | -0.67 | 1.04 |
|  | 50 | 1.66 | 1.00 | 4.07 | 0.33 | 2.67 |
|  | 500 | 0.46 | 0.27 | 1.17 | -0.23 | 0.50 |
| Rh1 | 5 | 1.50 | 0.33 | 1.66 | 1.00 | 1.50 |
|  | 50 | 1.66 | 1.00 | 2.71 | 0.67 | 2.35 |
|  | 500 | 0.64 | 0.03 | 1.17 | 0.23 | 0.90 |
| Rc | 50 | 2.97 | 1.33 | 1.93 | 1.67 | 1.97 |
|  | 500 | 0.98 | 0.40 | 1.41 | 0.57 | 1.04 |
|  | 5000 | 0.87 | 0.29 | 1.37 | -0.36 | 1.22 |
| Rd | 5 | 1.73 | 1.50 | 3.19 | 0.33 | 1.66 |
|  | 50 | 5.79 | -0.67 | 4.07 | 0.33 | 2.35 |
|  | 500 | 0.63 | 0.40 | 0.88 | 0.17 | 1.10 |
| Rb1 | 50 | 1.47 | 1.60 | 2.61 | 1.67 | 4.44 |
|  | 500 | 0.86 | 0.63 | 1.47 | 0.60 | 2.06 |
|  | 5000 | 0.86 | 0.55 | 1.29 | 0.51 | 1.83 |
| Rb2 | 50 | 1.93 | 1.63 | 3.60 | -0.90 | 2.58 |
|  | 500 | 1.30 | 0.30 | 1.76 | 0.63 | 2.29 |
|  | 5000 | 0.46 | 0.43 | 1.30 | 0.06 | 2.32 |
| Rh2 | 5 | 1.91 | 1.17 | 5.69 | -1.86 | 4.81 |
|  | 50 | 2.47 | 1.37 | 2.74 | 0.55 | 3.83 |
|  | 500 | 1.46 | 0.87 | 1.67 | 0.20 | 2.84 |
| Rg3 | 5 | 2.92 | 1.50 | 2.68 | 0.94 | 3.32 |
|  | 50 | 5.30 | 2.03 | 3.93 | -0.48 | 3.56 |
|  | 500 | 1.58 | 0.41 | 1.58 | 0.18 | 1.78 |

Table S3. Recovery and matrix effect of bioactive ginsenosides in plants (n = 6)

| Ginsenosides | Concentration (ng/mL) | Recovery (%) | | Matrix effect (%) | |
| --- | --- | --- | --- | --- | --- |
|  |  | Mean ± Standard Deviation | Relative standard deviation | Mean ± Standard Deviation | Relative standard deviation |
| Rg1 | 50 | 102.33±0.024 | 2.35 | 99.47±0.045 | 4.61 |
|  | 500 | 99.93±0.004 | 0.47 | 104.09±0.062 | 5.99 |
|  | 5000 | 99.14±0.026 | 2.62 | 101.18±0.030 | 3.01 |
| Re | 50 | 98.77±0.033 | 3.37 | 99.03±0.021 | 2.12 |
|  | 500 | 101.25±0.022 | 2.24 | 99.37±0.035 | 3.54 |
|  | 5000 | 103.07±0.063 | 6.11 | 100.47±0.016 | 1.61 |
| Rf | 5 | 97.00±0.078 | 8.12 | 98.00±0.066 | 6.83 |
|  | 50 | 99.03±0.025 | 2.61 | 100.65±0.048 | 4.85 |
|  | 500 | 99.87±0.006 | 0.61 | 99.45±0.011 | 1.20 |
| Rh1 | 5 | 100.28±0.020 | 2.05 | 99.58±0.016 | 1.64 |
|  | 50 | 100.37±0.0351 | 3.50 | 99.70±0.041 | 4.16 |
|  | 500 | 99.74±0.0103 | 1.03 | 99.54±0.008 | 0.84 |
| Rc | 50 | 98.90±0.057 | 5.79 | 99.04±0.036 | 3.64 |
|  | 500 | 101.16±0.019 | 1.88 | 99.94±0.029 | 2.96 |
|  | 5000 | 99.71±0.014 | 1.47 | 99.94±0.018 | 1.81 |
| Rd | 5 | 97.22±0.063 | 6.48 | 100.26±0.057 | 5.77 |
|  | 50 | 97.67±0.054 | 5.56 | 101.67±0.040 | 4.02 |
|  | 500 | 99.35±0.019 | 1.99 | 98.54±0.042 | 4.36 |
| Rb1 | 50 | 101.33±0.054 | 5.39 | 98.67±0.027 | 2.77 |
|  | 500 | 99.00±0.025 | 2.55 | 100.45±0.029 | 2.90 |
|  | 5000 | 99.84±0.018 | 1.89 | 98.90±0.017 | 1.78 |
| Rb2 | 50 | 99.88±0.060 | 6.07 | 100.40±0.043 | 4.34 |
|  | 500 | 99.23±0.030 | 3.08 | 98.20±0.051 | 5.21 |
|  | 5000 | 98.50±0.025 | 2.59 | 103.00±0.056 | 5.46 |
| Rh2 | 5 | 101.33±0.035 | 3.46 | 99.67±0.046 | 4.65 |
|  | 50 | 99.00±0.048 | 4.91 | 99.33±0.037 | 3.75 |
|  | 500 | 99.47±0.031 | 3.20 | 101.30±0.024 | 2.44 |
| Rg3 | 5 | 98.65±0.062 | 6.29 | 102.24±0.043 | 4.29 |
|  | 50 | 101.00±0.035 | 3.49 | 99.67±0.026 | 2.67 |
|  | 500 | 99.97±0.010 | 1.05 | 98.84±0.042 | 4.28 |
